# Frequency of pap smear among doctors: A pilot study

**DOI:** 10.12669/pjms.36.4.1651

**Published:** 2020

**Authors:** Lamia Yusuf

**Affiliations:** Dr. Lamia Yusuf, FCPS, MHPE. Associate Professor, Department of Gynaecology & Obstetrics, Rashid Latif Medical College, Lahore, Pakistan

**Keywords:** PAP Smear, Doctors, Prevalence, HPV Vaccination

## Abstract

**Background and Objective:**

Carcinoma of the cervix is one of the three leading causes of deaths among females worldwide. Pap smear is a simple and very cost-effective method to detect carcinoma of the cervix. The objective of our study was to determine the frequency of papsmear among doctors, so the alarming situation of sloppiness in the screening program can be highlighted.

**Methods:**

The interview-based survey was conducted; multiple questions were asked from the participants. It was a pilot study. Sixty doctors who were married (working in a teaching hospital) were recruited from Rashid Latif Medical College, Lahore from June 2018 - November 2018 and associated tertiary care teaching hospitals for the study. Ethical consideration was taken into account and secrecy of participants were maintained.

**Results:**

All data was entered in SPSS 21 and statistical analysis was done in terms of frequencies. Only 25% of doctors have a pap smear once in their life. Majority 75% of the doctors never have papsmear in their lifetime, few reasons were a shortage of time (27%) and shyness (5%). Regarding HPV vaccination 89% of the participants were willing to have HPV vaccination for their daughters.

**Conclusion:**

This study shows the poor implementation of the cancer screening programme in Pakistan. The general public should be informed about the benefits of HPV vaccination.

## INTRODUCTION

Cervical carcinoma is among one of the leading causes of maternal death due to malignancies. It is responsible for over twenty seven lac deaths, particularly in underdeveloped countries. The reason being a disparity between available health facilities. According to Badar et al.^1^ incidence of carcinoma of the cervix, although low in Pakistani women 4.1% as compared to western countries, but the late presentation of the patient is responsible for higher mortality rate. Cervical cancer is caused by persistent infection with human papillomavirus (HPV) type 16,18,31,33,4. The transmission mainly occurs via sexual activity, commonly through vaginal and anal intercourse. Cervical cancer develops slowly over time, start with HPV infection, which in some cases continues and advances to precancerous lesions that can cultivate into invasive cancer if ignored and untreated.^2^ The World Health Organization endorsements on target ages and frequency of cervical cancer screening affirm that screening should commence on women from 30 years or more. A 3-year interval can be considered in the age group 25-49 years, and screening is not necessary for women over 65 years.^3^

Screening asymptomatic women for precancerous lesions using the Papanicolaou (Pap) test results in an average drop of nearly 2.6% per year in cervical cancer mortality, in those countries with vigorous and dynamic health systems. Papanicolaou (Pap) smear is one of the most important screening tools for the early diagnosis of cervical cancer and the most effective preventive measures. Additional Cost-effective tools are available for the prevention and control of cervical cancer. the sensitivity of the pap smear in detecting a high-grade squamous intraepithelial lesion is 70%. Pap smear in association with HPV, DNA testing has a high sensitivity for early detection of precancerous disease.^4^

New technologies and approaches, including HPV vaccines, HPV tests, and a “screen and treat” method, have been developed and proven to effectively prevent cervical cancer. The cost-effectiveness of prevention strategies has been well recognized, showing that HPV vaccination coupled with screening is further cost-effective than either strategy alone. If promoted on a large scale, these new cost-effective interventions and approaches have the potential to accelerate reductions in cervical cancer mortality. It is rightly said “Prevention is better than cure”, HPV vaccination provides only prevention but for ongoing cervical diseases, routine screening should be continued in patients given HPV vaccination.^5^ The American Cancer Society (ACS) has recommended routine HPV vaccination primarily for females of age 11-12 years. ACS also recommended that girls ages 13-18 years should catch up who missed the opportunity to be vaccinated or who need to complete the vaccination series.^6^ National Cancer Control Programs are being implemented in various south Asian countries, including Pakistan, on World Health Organization recommendations. Education to increase awareness of cancer is essential where screening is lacking.^7^

The objective of this study was to determine the frequency of pap smear among doctors in this way, it may help to determine the prevalence of carcinoma cervix and also accentuate the importance of screening.

## METHODS

The participants of this study were recruited from Rashid Latif Medical College, Lahore and associated tertiary care teaching hospitals. It was carried out from June 2018 - November 2018. All participants were medical graduates from recognized medical colleges/ universities. Almost all of the participants are working in senior positions. An interview-based survey was conducted to determine the frequency of Pap smear among doctors. It was a pilot study and 60 doctors who were married were included.^8^

Subjects were recurited through convinent sampling method. The information was collected on a questionnaire, desigened to assess, familiarity, conducts and thoughts of doctors towards this screening test. The questionnaire was developed based on literature review and validate from consultant gynaecologist and medical educationists. Permission was obtained from the ethical review committee (Ref. No. IRB/2018/004) and participants gave verbal consent. Privacy of participants was ensured.

**Fig.1:**
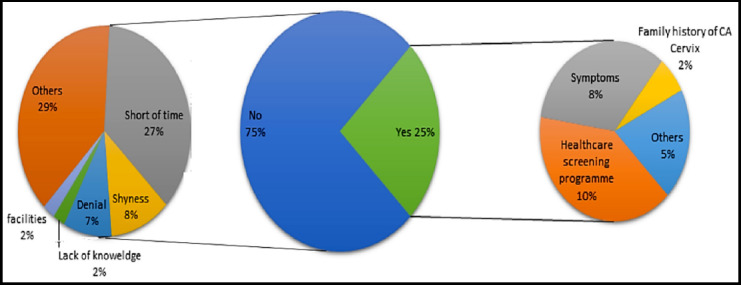
Percentage of participants having Pap Smear.

**Table-I:** Participants responses to the utilization of papsmear.

PS	Reason	PS
Yes	Healthcare screening program	6(10.17%)
	Symptoms	5(8.47%)
	Family history of CA Cervix	1(1.69%)
	Others	3(5.08%)
No	Short of time	16(27.12%)
	Lack of knowledge	1(1.69%)
	Denial	4(6.78%)
	facilities	1(1.69%)
	Shyness	5(8.47%)
	Others	17(28.81%)

## RESULTS

All data was entered in SPSS 21 and statistical analysis was done in terms of frequencies. In this, the mean age of participants was 36.59±8.49 years whereas the minimum age of the participant was 26 and the maximum age was 65 years.

Among the participants, papsmear was performed by only 15 participants (25.42%).

When asked to whom you will suggest pap smear 16(27.1%) replied every patient, 26(44.1%) replied those required and 17(28.8%) did not reply.

## DISCUSSION

This pilot study was conducted to determine the prevalence of Pap smear among doctors, the purpose was to highlight the frightening situation of neglect in the screening of cervical diseases. Pakistan is an underdeveloped country where the late presentation of patients suffering from cancer is a cause of mortality.

The results of this study are very frightening as only, 25% of the doctors had a pap smear. The most common reason for having their smear is as a routine health screening test, though only 10% of the doctors follow regular health screening program, Seventy-five (75)% have never had a pap smear once in their life. The majority of the participants gave a reason for not having papsmear was a shortage of time 27%, which is apparent from the lifestyle and busy schedule of doctors. Only 1% said that they lack knowledge about a pap smear. This study shows that pap smear utilization is very low despite high degree, knowledge and awareness.^9^ Doctors being highly educated are supposed to be knowledgeable about essential screening programs. In Pakistan according to PM&DC curriculum, papsemar is being taught in different arrangements from the third year to final year MBBS during pathology and gynae obstetrics classes. According to Redhwan et al, the more knowledge women have about pap smear, the more they are particular about having a pap smear.^10^ Same findings are discussed in a study by Sevil S et al.^10^ but this is contrary to findings described in our study. This not only raises questions on the health screening program but also upon the quality of medical education given in our country. Though our study shows that 68% of the doctors have information about pap smear screening, but knowledge is not the sole factor of health practices of individuals a similar low uptake of papsmear is seen among female health care providers in another study.^11^ Shyness (5%) denial to perform pap smears and few other reasons, are the factors why the prevalence of Pap smear is so low in this pilot study.^12^

In this pilot study, 93% of the participants affirmed that they have heard about HPV VACCINATION and 89.9% were willing to vaccinate their daughters. These results were comparable to the study conducted by Ilter E et al.^13^ This vaccine has sustained long-term vaccine efficacy against HPV infection and cervical cancer. In a conservative country like Pakistan talking to patients about sex, multiple sexual partners and sex-related infections are very difficult. So while asking questions while taking a history from a woman (patient) who present with the cervical premalignant condition, one has to be very careful and tactfully deal with the scenario. Though Being a Muslim society the majority of couples have safe sex practices but still, we have a class of people where these problems of multiple sexual partners and other social and moral dilemmas are common. Prophylactic vaccinations against HPV infection bear the potential to reduce the burden of cervical cancer and have proven to be cost-effective when offering to women before having an infection. It is good that vaccination should be given before entering into sexual activity. Mothers play an important role to decide about their daughters to have HPV vaccinations. Their concern about the acceptance of having vaccination, advantages, disadvantages, and cost of this vaccination should be addressed by health care providers.^14^ The health care provider should be trained to discuss these issues with parents and girls. They should reflect this awareness to the public,^15^,^16^ thus enhancing the familiarity and inclination of the public toward the pap smear screening program and early vaccination of their daughters.^17^,^18^ IT is recommended to carry out a cross-sectional study on a large scale, so as to achieve the aims of the study.

**Table-II:** Responses of participants.

	Yes	No	Total	No Response
Do you know regular Schedule of pap smear	40(67.8%)	16(27.1%)	56(94.9%)	3(5.1%)
Taught pap smear	44(74.6%)	13(22.0%)	57(96.6%)	2(3.4%)
Does your institute have regular screening program	18(30.5%)	40(67.8%)	58(98.3%)	1(1.7%)
Given Advise	17(28.8%)	38(64.4%)	55(93.2%)	4(6.8%)
Have you suggested to any other	40(67.8%)	15(25.4%)	55(93.2%)	4(6.8%)
Will you follow it if pap smear screening Offered at your institution	51(86.4%)	7(11.9%)	58(98.3%)	1(1.7%)
Have you heard about HPV Vaccination	55(93.2%)	4(6.8%)		
Would you vaccinate your Daughters or other family members	53(89.8%)	5(8.5%)		1(1.7%)

## CONCLUSIONS

The result of this pilot study (although very limited) implies a review of our health care system. The regularity of practising papsmear is unfortunately very low among doctors. Our young girls should get the benefit of HPV vaccination, thus ensuring women empowerment regarding their health issues.
